# High-dose vitamin C ameliorates cardiac injury in COVID-19 pandemic: a retrospective cohort study

**DOI:** 10.18632/aging.203503

**Published:** 2021-09-09

**Authors:** Guozhi Xia, Bowen Qin, Chaoran Ma, Yaowu Zhu, Qiangsun Zheng

**Affiliations:** 1 Department of Cardiology, The Second Affiliated Hospital of Xi'an Jiaotong University, Xi'an 710004, Shaanxi Province, China; 2 National-Local Joint Engineering Research Center of Biodiagnostics and Biotherapy, The Second Affiliated Hospital of Xi'an Jiaotong University, Xi'an 710004, Shaanxi Province, China; 3 Department of Nutritional Sciences, Pennsylvania State University, University Park, PA 16802, USA; 4 Department of Laboratory Medicine, Tongji Hospital of Huazhong University of Science and Technology, Wuhan 430030, Hubei Province, China

**Keywords:** vitamin C, inflammation, cardiac injury, COVID-19

## Abstract

Background: Cardiac injury is common and associated with poor clinical outcomes in COVID-19. Data are lacking whether high-dose intravenous vitamin C (HIVC) could help to ameliorate myocardial injury in the pandemic.

Methods: The retrospective cohort study included consecutive severe and critically ill COVID-19 patients with cardiac injury receiving symptomatic supportive treatments alone or together with HIVC. Troponin I and inflammatory markers were collected at admission and day 21 during hospitalization from the electronic medical records.

Results: The patients (n = 113) were categorized into the ameliorated cardiac injury (ACI) group (n = 70) and the non-ameliorated cardiac injury (NACI) group (n = 43). Overall, fifty-one (45.1%) patients were administered with HIVC, the percentages of patients with HIVC were higher in the ACI group than those in the NACI group. Logistic regression analysis revealed that HIVC was independently associated with the improvement of myocardial injury. Further analysis showed that inflammatory markers levels significantly decreased at day 21 during hospitalization in patients with HIVC therapy compared to those administered with symptomatic supportive treatments alone. Meanwhile, similar results were also observed regarding changes in inflammatory markers levels from baseline to day 21 during hospitalization in the patients treated with HIVC.

Conclusions: HIVC can ameliorate cardiac injury through alleviating hyperinflammation in severe and critically ill patients with COVID-19.

## INTRODUCTION

Coronavirus disease 2019 (COVID-19) caused by a novel coronavirus named SARS-CoV-2 poses a worldwide healthcare issue with the increasing number of infected individuals [1]. The result of a recent report has shown that myocardial damage is a very common issue with increased risk of poor clinical prognosis in the course of severe cases [2]. It is well established that cytokines play an essential role in the modulation of systemic inflammatory response to coronavirus infections [3]. A growing body of evidence revealed that patients with severe illness might have a high inflammatory burden, which predicted adverse clinical events in individuals suffering from COVID-19 infection; particularly, in patients in case of cardiac injury [2, 4]. Crucial evidence of our previous results indicated that inflammatory markers were dramatically increased, indicating an independent link in the development of myocardial damage in the pandemic [5]. Therefore, myocardial injury may be a consequence of hyperinflammation among patients suffering from COVID-19 pneumonia.

Given the background, it is urgently needed to study new therapeutic options to suppress hyperinflammation for ameliorating cardiac injury and reducing fatality. At present, managing COVID-19 is challenging as there is lack of antiviral agents against SARS-CoV-2. Symptomatic supportive treatment is still the current main therapeutic strategy in the pandemic. Clinical efforts focus on repurposing already approved drugs for treatment of the disease. Vitamin C is significant to human body and potentiates the ameliorative effect of inflammatory-induced tissue damage. Additionally, the use of intravenous vitamin C arises from experimental evidence of its anti-inflammatory properties [6]. A recent study has demonstrated the clinical efficacy of high-dose intravenously administered vitamin C (HIVC) in terms of reducing fatality in patients with sepsis [7]. Thus, we hypothesized that HIVC could help to improve myocardial damage by mitigating hyperinflammation in a subset of COVID-19 cases. Data are lacking whether HIVC could work as an adjunctive medical treatment for ameliorating myocardial damage in the pandemic. Hence, the aim of the study reported here was to elucidate the therapeutic efficacy of HIVC on cardiac injury among COVID-19 pneumonia in severe and critically ill condition.

## MATERIALS AND METHODS

### Study design and subjects

A retrospective, observational cohort analysis was carried out from February 1 to March 10, 2020. The study subjects consisted of COVID-19 pneumonia with myocardial damage who consecutively admitted to Tongji Hospital of Huazhong University of Science and Technology (Wuhan, China), and were diagnosed as positive for SARS-CoV-2 infection by performing RT-PCR assay on samples from nasal and pharyngeal swab, and were in severe and critically ill condition. Identification of disease condition was achieved in accordance with the criteria in Chinese management guidelines for COVID-19 on February 4, 2020 (trial version 5.0) [8]. The exclusion criteria were listed as follows: (i) patients without complete medical records; (ii) those who were not confirmed to be infected by SARS-CoV-2 up to March 24, 2020; (iii) individuals who lacked determinations of high-sensitivity troponin I (hs-cTnI) and parameters of inflammation on admission and day 21 during hospitalization.

The study complied with the ethical principles of the World Medical Association’s Declaration of Helsinki and received the ethical approval from the Medical Ethics Committee of the hospital. The oral, not written informed consents were obtained from patients underwent HIVC therapy owing to observational nature of the research and isolation precautions of the rapidly evolving pandemic.

### Data sources

The screening exam comprised patient’s demographics details and baseline clinical characteristics available in the hospital’s electronic medical records system for analysis. The following information was recorded for each patient: age, gender, smoking status, pulse, blood pressure, comorbidity (i.e., hypertension, coronary heart disease, and diabetes), time from illness onset to admission, as well as presenting clinical symptoms. Simultaneously, we collected the laboratory examinations on complete blood counts, haemoglobin, serum biochemical indicators, N-terminal pro-B-Type natriuretic peptide (NT-proBNP) at baseline. The parameters of inflammation including high-sensitivity C-reactive protein (hs-CRP), interleukin (IL)-6, IL-8, tumor necrosis factor (TNF)-α, and hs-cTnI were obtained at admission and repeated day 21 during hospitalization.

hs-cTnI, NT-proBNP and inflammatory markers determinations were conducted in the central laboratory of Union Hospital. A blood sample was acquired at admission (defined as day 1) and finally beyond day 20 to discharge (defined as day 21 during hospitalization). All procedures were carried out following standard operating procedures.

### Treatment regimen

On admission, all subjects were initiated on symptomatic supportive treatments based on the requirements of each individual patient. As suggested in the guideline, [8] symptomatic supportive treatments consisted of antiviral, antibiotics, corticosteroids, immunoglobulin, biologics, mechanical ventilation, rennin-angiotensin system inhibitors, nutritional supplements for myocardium, renal replacement therapy, and antidiabetic therapy prescribed by our medical group according to the hospital protocol, unless contraindicated. Besides symptomatic supportive treatments, a subset of patients with verbal consents also received administration of HIVC infusion as adjuvant therapy during the first 24 hours period after admission. The HIVC treatment protocol was administered as reported previously by our group [9]. Briefly, vitamin C was administered intravenously at the excess dosage of 100 mg/kg every 6 hours for 1 day followed by 100 mg/kg every 12 hours for additional 5 days during hospitalization.

### Definitions

The clinical spectrum of COVID-19 ranges from mild, common, severe to critically ill cases. Severe illness was considered in the presence of one or all three of the following criteria: (i) dyspnea with respiratory rate ≥ 30 breaths/min; (ii) arterial oxygen saturation ≤ 93% at a rest state; (iii) the ratio of arterial partial pressure of oxygen/fractional inspired oxygen < 300 mm Hg [8]. Patients who met one of the following conditions were considered to have critically ill pneumonia: (i) respiratory failure and mechanical ventilation required; (ii) sepsis shock; (iii) multiorgan failure [8]. The efficacy evaluation of HIVC therapy on myocardial injury was performed through screening changes on concentrations of hs-cTnI and inflammatory parameters from baseline to day 21 during hospitalization. Myocardial damage was evidenced by blood levels of hs-cTnI > the 99th percentile upper reference limit (26.2 pg/mL) [2, 10]. Ameliorated cardiac injury (ACI) was diagnosed on the basis of the serum levels of hs-cTnI at day 21 during hospitalization below 26.2 pg/mL; non-ameliorated cardiac injury (NACI) was adjudicated on the serum levels of hs-cTnI at day 21 during hospitalization above 26.2 pg/mL, or those of baseline. Hyperinflammation was determined by the elevated levels of inflammatory markers [11].

### Statistical analysis

We expressed continuous variables as median with interquartile range (IQR, 25-75th percentile) or categorical variables as number with percentage to characterize the study cohort. When continuous variables conformed to a normal distribution, 2-sample independent group *t* test was applied to analyze differences between the ACI group and the NACI group; otherwise, the Mann-Whitney U test was conducted. The assumption of normal distribution of the data was determined using the Shapiro-Wilk test. Proportions for categorical variables were compared using chi-square test or Fisher's exact test where appropriate. The independent association between medications and cardiac injury was studied using univariate and multivariate logistic regression analysis accounting for HIVC as well as other symptomatic supportive treatments. The results were described as odds ratio (OR) and 95% confidence interval (CI). A forest plot was created base on logistic regression analysis results. Hypothesis tests were performed using two-tailed test with *P* value of < 0.05 indicating statistical significance. All statistical analysis was performed using SPSS (version 16.0) and GraphPad Prism 5.0.

## RESULTS

### Baseline characteristics and laboratory findings

The detailed analysis of baseline characteristics in our study cohort was described in Table 1. A total of 113 confirmed COVID-19 cases were included in the study: 70 in the ACI group and 43 in the NACI group. The median age was 68 years old, and the male ratio was 52/61 (46.0%). There were no significant between-group differences for demographics and baseline clinical characteristics. Meanwhile, similar results were also observed between the two groups with respect to inflammatory markers levels at baseline.

**Table 1:** Baseline characteristics and laboratory findings.

**Characteristics**	**ACI (n = 70)**	**NACI (n = 43)**	***P* value**
Demographics			
Age, years	68 (59-77)	71 (63-77)	0.557
Male gender, n (%)	32 (45.7)	20 (46.5)	0.934
Current smoker, n (%)	17 (24.3)	15 (34.9)	0.225
Heart rate, beats per min	90 (82-107)	91 (78-110)	0.946
Systolic blood pressure, mm Hg	136 (122-146)	134 (121-146)	0.679
Diastolic blood pressure, mm Hg	79 (76-87)	77 (70-88)	0.329
Cardiovascular comorbidities			
Hypertension, n (%)	26 (37.1)	23 (53.5)	0.089
Coronary heart disease, n (%)	10 (14.3)	12 (27.9)	0.076
Diabetes, n (%)	13 (18.6)	14 (32.6)	0.090
Time from illness onset to admission, days	9 (6-13)	11 (7-15)	0.078
Main clinical symptoms at onset of illness			
Fever (temperature > 37.5° C), n (%)	56 (80.0)	32 (74.4)	0.488
Cough, n (%)	38 (54.3)	24 (55.8)	0.874
Sputum, n (%)	13 (18.6)	9 (20.9)	0.758
Dyspnea, n (%)	20 (28.6)	8 (18.6)	0.233
Fatigue, n (%)	17 (24.3)	11 (25.6)	0.877
Diarrhea, n (%)	13 (18.6)	6 (14.0)	0.524
Laboratory findings			
Leukocyte counts, cells × 10^9^/L	9.15 (5.84-11.16)	9.27 (7.53-12.73)	0.176
Lymphocyte counts, cells × 10^9^/L	0.65 (0.51-0.97)	0.55 (0.41-1.01)	0.917
Erythrocyte counts, cells × 10^12^/L	3.91 (3.61-4.48)	4.04 (3.49-4.54)	0.753
Platelet counts, cells × 10^9^/L	217 (149-260)	196 (134-240)	0.317
Haemoglobin, g/L	126 (109-141)	127 (101-141)	0.465
Albumin, g/L	31.5 (29.5-34.9)	31.8 (28.7-34.6)	0.520
Alanine aminotransferase, U/L	30 (16-41)	25 (17-36)	0.429
Aspartate aminotransferase, U/L	37 (25-57)	34 (24-55)	0.761
Urea nitrogen, mmol/L	7.5 (5.3-9.2)	7.5 (5.3-9.5)	0.390
Serum creatinine, μmol/L	85 (61-99)	84 (72-113)	0.118
NT-proBNP, pg/mL	961 (362-2424)	790 (307-2828)	0.743
Inflammatory parameters			
hs-CRP, mg/L	66.3 (27.6-103.8)	70.4 (23.0-112.6)	0.645
IL-6, pg/mL	67.4 (33.4-124.1)	77.2 (26.9-183.8)	0.709
IL-8, pg/mL	38.8 (18.8-90.9)	33.8 (19.4-67.0)	0.460
TNF-α, pg/mL	11.6 (7.9-20.0)	12.9 (8.4-19.9)	0.574

Data are median with interquartile range (IQR, 25-75th percentile) values or number (n) with percentage (%) of patients. 2-sample independent group *t* test, Mann-Whitney U test or chi-square test were performed between the ACI and NACI groups, as appropriate. *P* < 0.05 was considered statistically significant. Abbreviations: ACI, ameliorated cardiac injury; NACI, non-ameliorated cardiac injury; NT-proBNP, N-terminal pro-B-Type natriuretic peptide; hs-CRP, high-sensitivity C-reactive protein; IL, Interleukin; TNF-α, Tumor necrosis factor-α.

**Figure 1:**
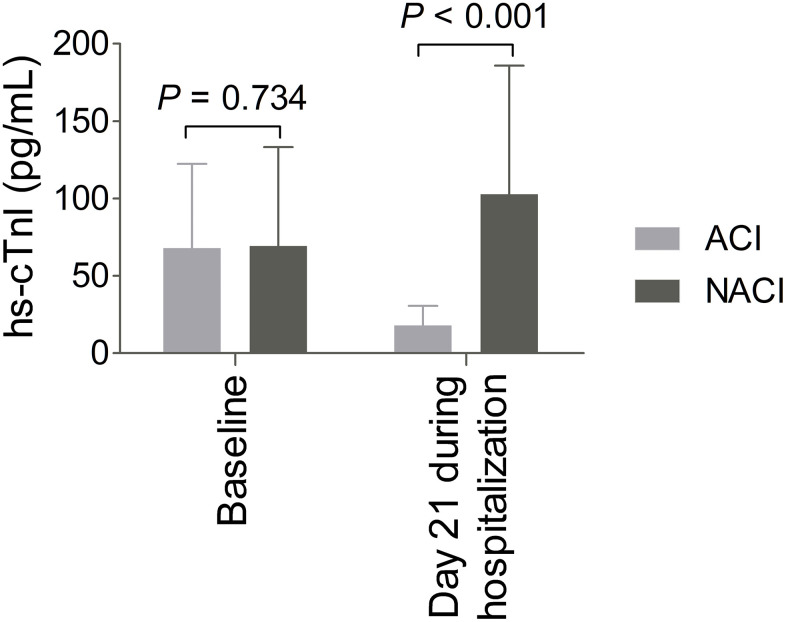
**Changes in levels of high-sensitivity troponin I (hs-cTnI) from baseline to day 21 during hospitalization.** The levels of hs-cTnI displayed no significant difference at baseline; whereas the hs-cTnI levels at day 21 during hospitalization appeared significantly lower in the ameliorated cardiac injury (ACI) group (n = 70) than those in the non-ameliorated cardiac injury (NACI) group (n = 43).

As shown in Figure 1, the serum levels of hs-cTnI displayed no significant difference between the ACI group and the NACI group at baseline [43.7 (30.9-83.3) pg/mL vs 51.4 (33.1-91.8) pg/mL, *P* = 0.734]. In contrast, the concentrations of hs-cTnI at day 21 during hospitalization appeared significantly lower in the ACI group compared with the NACI group [17.6 (12.2-22.9) pg/mL vs 76.5 (49.2-126.5) pg/mL, *P* < 0.001].

### Effect of HIVC on cardiac injury

Of the 113 hospitalized patients in our study, 62 (54.9%) cases were administered with symptomatic supportive treatments alone and 51 (45.1%) received administration with HIVC in addition to symptomatic supportive treatments. The percentages of subjects with HIVC therapy were 52.8% (n = 37) in the ACI group and 32.5% (n = 14) in the NACI group, respectively; and the proportions were significantly different (χ^2^ = 4.432, *P* = 0.035; Table 2). Likewise, the percentages of patients receiving biologics (χ^2^ = 4.336, *P* = 0.049), mechanical ventilation (χ^2^ = 5.230, *P* = 0.029), and renal replacement therapy (χ^2^ = 4.484, *P* = 0.045) were also statistically significant between the two cohorts (Table 2).

**Table 2:** Medications during hospitalization.

**Medications**	**ACI (n = 70)**	**NACI (n = 43)**	***P* value**
Antiviral, n (%)	59 (84.3)	34 (79.1)	0.481
Antibiotic, n (%)	50 (71.4)	33 (76.7)	0.534
Corticosteroid, n (%)	28 (40.0)	16 (37.2)	0.768
Immunoglobin, n (%)	16 (22.9)	10 (23.3)	0.961
Biologics, n (%)	10 (14.3)	1 (2.3)	0.049
Mechanical ventilation, n (%)	19 (27.1)	4 (9.3)	0.029
Rennin-angiotensin system inhibitors, n (%)	14 (20.0)	9 (20.9)	0.905
Nutritional supplements for myocardium, n (%)	27 (38.6)	18 (41.9)	0.729
Renal replacement therapy, n (%)	13 (18.6)	2 (4.7)	0.045
Antidiabetic, n (%)	11 (15.7)	10 (23.3)	0.317
HIVC	37 (52.8)	14 (32.5)	0.035

Data are expressed as number (n) with percentage (%). *P* values for comparison between ACI and NACI group were calculated based on chi-square test or Fisher’s exact test where appropriate. *P* < 0.05 was considered statistically significant. Abbreviations: HIVC, high-dose intravenous vitamin C; ACI, ameliorated cardiac injury; NACI, non-ameliorated cardiac injury.

**Figure 2:**
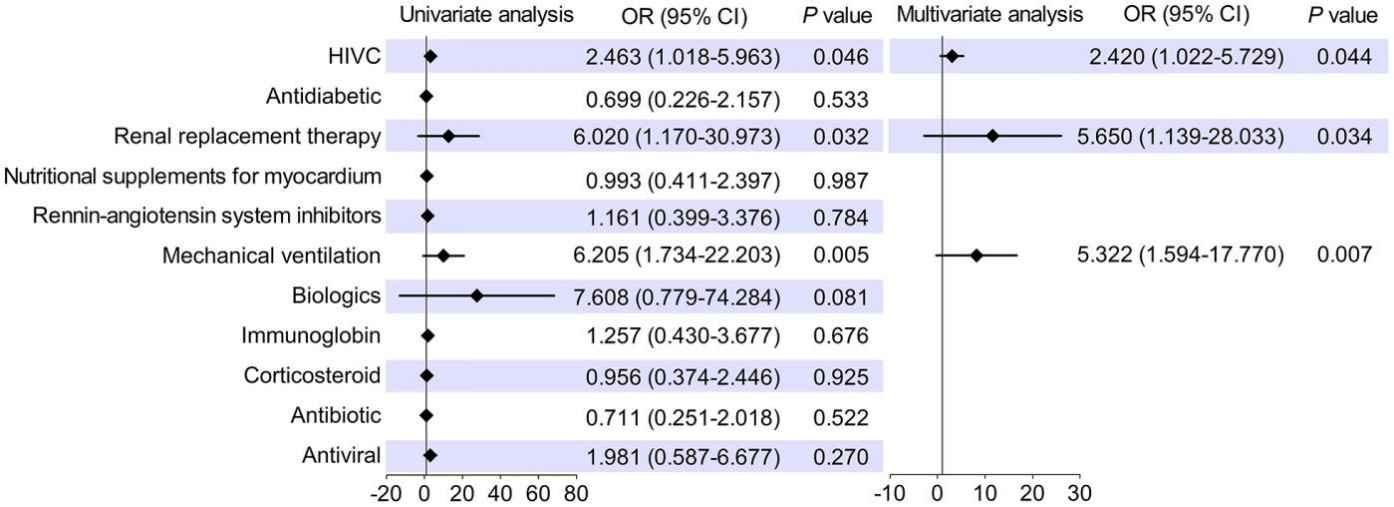
**Forest plot displayed high-dose intravenous vitamin C (HIVC) was associated with ameliorated cardiac injury independent of other medications.** OR, odds ratio; CI, confidence interval.

To investigate the independent effect of HIVC on cardiac injury in more detail, logistic regression analysis was performed with variables entered in the model including HIVC and other medications during hospitalization. The result revealed that administration of HIVC infusion was correlative with the improvement of cardiac injury (*OR* 2.420, 95% CI 1.022-5.729, *P* = 0.044; Figure 2) independent of mechanical ventilation (*OR* 5.322, 95% CI 1.594-17.770, *P* = 0.007) and renal replacement therapy (*OR* 5.650, 95% CI 1.139-28.033, *P* = 0.034). Thus, HIVC seemed to show a beneficial effect in improving myocardial damage among patients with SARS-CoV-2 infection in severe and critically ill condition.

### Effect of HIVC on hyperinflammation

**Figure 3:**
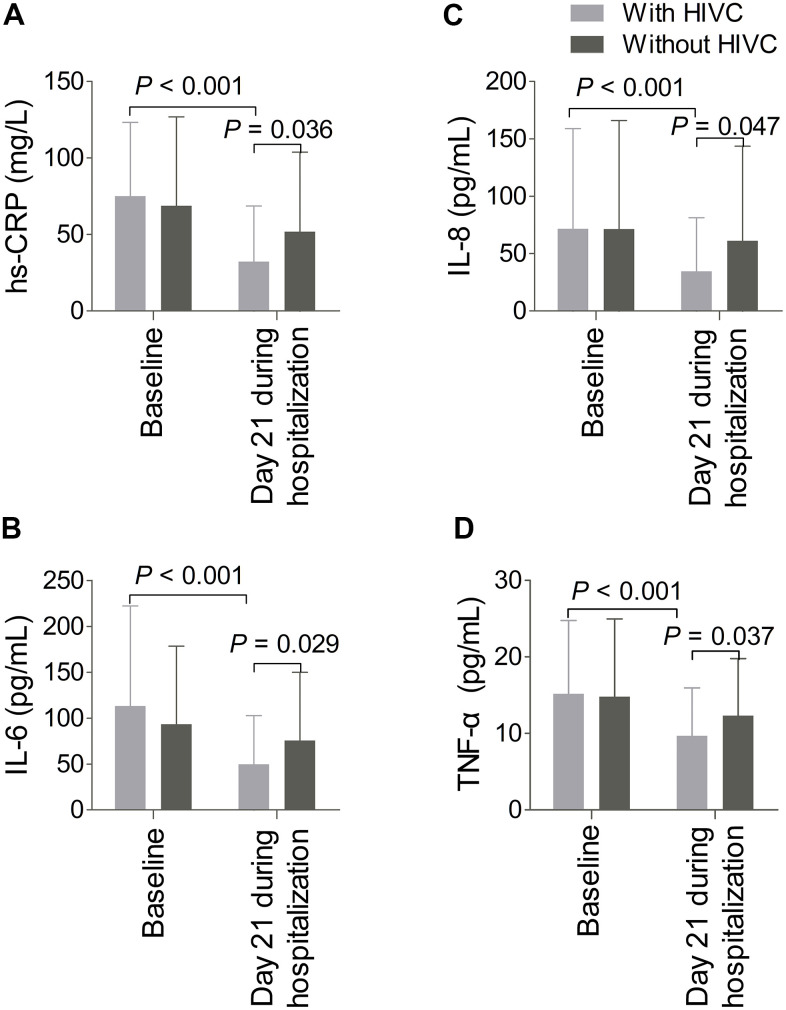
**Changes in inflammatory markers levels from baseline to day 21 during hospitalization.** The levels of high-sensitivity C-reactive protein (hs-CRP, **A**), Interleukin-6 (IL-6, **B**), IL-8 (**C**), and tumor necrosis factor-α (TNF-α, **D**) at day 21 during hospitalization in patients administered with high-dose intravenous vitamin C (HIVC, n = 51) significantly decreased compared with those receiving symptomatic supportive treatments alone (n = 62). In addition, changes of the inflammatory markers levels displayed a tendency of significant decrease at day 21 during hospitalization related to baseline in patients underwent HIVC therapy.

We subsequently investigated changes in parameters of inflammation from admission to day 21 during hospitalization. As described in Figure 3, regarding the levels of inflammation parameters on admission, no statistical significances were found between the two cohorts of patients underwent HIVC administration (n = 51) or symptomatic supportive treatments alone (n = 62) (hs-CRP, *P* = 0.303; IL-6, *P* = 0.460; IL-8, *P* = 0.676; TNF-α, *P* = 0.552). However, the levels of inflammation parameters at day 21 during hospitalization among cases receiving HIVC therapy significantly decreased compared to those administered with symptomatic supportive treatments alone (hs-CRP, *P* = 0.036; IL-6, *P* = 0.029; IL-8, *P* = 0.047; TNF-α, *P* = 0.037). Furthermore, changes in inflammatory markers levels displayed a tendency of significant decrease at day 21 during hospitalization related to baseline in patients receiving HIVC therapy (all *P* < 0.001).

## DISCUSSION

The pandemic of COVID-19 continues to spread worldwide with a serious threat to human life, there are not yet effective treatments that target SARS-CoV-2, it is urgent to find new preventive and therapeutic strategies as soon as possible to potentially protect from the virus or to alleviate its effects once caught. One such medication that is being touted in the media is HIVC. However, the efficiency of HIVC in the management of SARS-CoV-2 infection is still controversial [12, 13]. In this study, our endeavor had focused on HIVC that targeted hyperinflammation leading to cardiac injury. Our choice of the agent was based on the fact that the therapeutic approach was readily available for clinical use, and HIVC has been shown to provide significant cardioprotective effect in a variety of disease states [14, 15]. There are also indications that HIVC administration may be potential benefit in the course of critical illness such as sepsis, in which the hyperinflammation is activated and leads to subsequent development of multiple organs dysfunction. However, HIVC can effectively attenuate this process. Moreover, vitamin C can help to reduce lung damage by preventing the activation of pro-inflammatory cytokines [16]. Although there is no universally adopted dosage definition, we recommended intravenous administration of a 6-day course of 100 mg/kg every 6 hours for 1 day followed by 100 mg/kg every 12 hours for additional 5 days as the optimum dosage of vitamin C therapy as described in the recently published randomized controlled trial to study sepsis-related severe acute respiratory failure [7].

There is a high prevalence of myocardial injury in the form of elevation in hs-cTnI at high risk for severe disease and death [10]. A large body of evidence suggests cardiac injury is an independent predictor for all-cause death in the cases of critically ill COVID-19 infection during hospitalization [10, 17, 18]. We performed an analysis of the current data to investigate whether HIVC may help to ameliorate myocardial damage in subjects who were in severe and critically ill situation. In our analysis, fortunately, the percentages of subjects receiving HIVC therapy were 52.8% in the ACI group and 32.5% in the NACI group, respectively, the difference was statistically significant, indicating the significant amelioration in cardiac injury can be explained with HIVC treatment. Furthermore, it was found from logistic regression analysis results that HIVC therapy was in close relationship with improvement of myocardial damage independent of other medications (i.e., mechanical ventilation, renal replacement therapy). Therefore, all the above observations supported the issue of clinical efficacy of HIVC on cardiac injury. The properties for cardioprotective effect of HIVC had been suggested by the fact that HIVC showed a positive impact on recovery from cardiac injury in this study. To reduce the risk of cardiac injury, it is recommended that HIVC is administered if possible and continues for a short period of time. However, evidence seems to be conflicting as a recently published a pilot trial showed no significant clinical benefit in the improvement of cardiac injury [13]. The trial investigated myocardial injury only after 3 days and 7 days of treatment, it was thus uncertain whether HIVC may have an ameliorative effect on cardiac involvement and avoid the deleterious consequences in SARS-CoV-2 infection at day 21 in the course of hospitalization.

To better understand the cardioprotective mechanism of HIVC, we assessed the impact of HIVC therapy on inflammatory markers levels in the subjects. We measured inflammatory markers levels of the cases underwent symptomatic supportive treatments alone or in addition to HIVC at admission and day 21 during hospitalization. The results indicated that patients administered with HIVC exhibited a significant decrease of concentrations of inflammation parameters compared with those receiving symptomatic supportive treatments alone. In addition, the changes in inflammation parameters levels displayed a tendency of significant decrease at day 21 during hospitalization related to baseline in patients treated with HIVC. On all these counts, we demonstrated the elevated levels of inflammatory markers that might result in hyperinflammation significantly decreased in patients administered with HIVC, which indicated that HIVC may be helpful in improving cardiac injury through mitigating hyperinflammation. As shown by the data available, COVID-19 frequently encounters conditions in which hyper-inflammatory response characterized by a cytokine storm results from an exaggerated response of the systematic inflammation in patients infected with SARS-CoV-2 [17]. Moreover, this overwhelming inflammatory stress contributes to the development of myocardial damage [17]. Cardiac injury is frequently encountered in the critically ill cases suffering from SARS-CoV-2 infection, which has been thought to be followed by a secondary hyperinflammation [10]. Herein, we speculated that hyperinflammation may be responsible for the pathological damage of myocardium. First, hyperinflammation can lead to ischemia of myocardium. In a pathology study, the local exacerbated inflammation destabilizes coronary atherosclerotic plaques which can result in increased risk of plaque rupture, and reducing coronary blood flow. In this respect, it also renders lesions more thrombogenic, a pro-thrombotic state which predispose to ischemia and subsequent myocardial damage [18]. Second, the procoagulant activity is enhanced in the case of hyperinflammatory course. Additionally, the active inflammatory mediators induce further coronary microvascular thrombosis, especially in the setting of underlying cardiovascular disease [19]. Third, the cytokines storm can contribute to diffuse endothelial injury, which leads to the formation of reactive oxygen and associated reduction of nitric oxide. Also, oxidative stress cause apoptosis or necrosis of myocardial cells [20]. Taken together, hyperinflammation is an important causative agent of the development and progression of myocardial injury in SARS-CoV-2 infection pneumonia.

### Limitations

Limitations to the present study include a single center experience with a relatively small sample size. It was not possible to perform a valid calculation of the sample size due to the critical condition of the ongoing pneumonia in our hospital. Also, the study was a nonrandomized observational study and hence suffered from potential selection and ascertainment biases. The determinations of parameters of inflammation should be very detailed at additional time points during and following treatment, but we only collected hs-CRP, IL-6, IL-8, and TNF-α at baseline and day 21 during hospitalization, it was thus uncertain whether HIVC may have an effect on other inflammatory markers levels. Furthermore, medications such as corticosteroids usage may affect levels of inflammatory markers. Importantly, we had no standardized protocol for HIVC, the dosage of HIVC used in this study may be insufficient for optimal care of SARS-CoV-2 infection. Further research on vitamin C dosages and longer administration time may need optimization.

## CONCLUSIONS

In this retrospective cohort study, our analysis of data from the patients treated with HIVC along with our tests of inflammatory markers showed the novel information that HIVC therapy may be potential benefit in ameliorating myocardial injury by alleviating hyperinflammation in the form of elevated levels of hs-CRP, IL-6, IL-8, and TNF-α. Therefore, our study supports the hypothesis that HIVC can ameliorate myocardial damage by alleviating hyperinflammation, it has shown promise as an adjunct therapeutic strategy for myocardial injury in the clinical management of COVID-19 pneumonia.
